# Natural Guided Genome Engineering Reveals Transcriptional Regulators Controlling Quorum-Sensing Signal Degradation

**DOI:** 10.1371/journal.pone.0141718

**Published:** 2015-11-10

**Authors:** Abbas El Sahili, Anthony Kwasiborski, Nicolas Mothe, Christophe Velours, Pierre Legrand, Solange Moréra, Denis Faure

**Affiliations:** 1 Institute for Integrative Biology of the Cell (I2BC), CNRS, CEA, Univ. Paris-Sud, Université Paris-Saclay, 91198 Gif-sur-Yvette Cedex, France; 2 Synchrotron SOLEIL, L’Orme des Merisiers, Saint Aubin BP48, Gif-sur-Yvette 91198, France; Shanghai Jiao Tong University, CHINA

## Abstract

Quorum-quenching (QQ) are natural or engineered processes disrupting the quorum-sensing (QS) signalling which controls virulence and persistence (e.g. biofilm) in numerous bacteria. QQ involves different enzymes including lactonases, amidases, oxidases and reductases which degrade the QS molecules such as N-acylhomoserine lactones (NAHL). *Rhodococcus erythropolis* known to efficiently degrade NAHL is proposed as a biocontrol agent and a reservoir of QQ-enzymes for biotechnology. In *R*. *erythropolis*, regulation of QQ-enzymes remains unclear. In this work, we performed genome engineering on *R*. *erythropolis*, which is recalcitrant to reverse genetics, in order to investigate regulation of QQ-enzymes at a molecular and structural level with the aim to improve the QQ activity. Deep-sequencing of the *R*. *erythropolis* enhanced variants allowed identification of a punctual mutation in a key-transcriptional factor QsdR (Quorum sensing degradation Regulation) which regulates the sole QQ-lactonase QsdA identified so far. Using biophysical and structural studies on QsdR, we demonstrate that QQ activity can be improved by modifying the regulation of QQ-enzymes degrading QS signal. This modification requiring the change of only one amino-acid in a transcriptional factor leads to an enhanced *R*. *erythropolis* in which the QS-signal degradation pathway is strongly activated.

## Introduction

Anti-virulence paradigm sustains the development of treatments which are alternative or complementary to antibiosis-based agents [1]. The regulatory pathways such as quorum-sensing (QS) which control bacterial behaviors are attractive targets of anti-virulence treatments [2]. In numerous bacteria, QS-signals are master regulators of a wide variety of behaviors (secretion of virulence factors, motility, horizontal gene transfer, biofilm development) which contribute to adaptation, proliferation and aggressiveness [3,4] The natural or engineered processes which disturb QS are called quorum-quenching [5]. Quorum-quenching (QQ) strategies encompass several molecular actors: chemical compounds (called QS-inhibitors) which inhibit synthesis, transport or perception of the QS-signals, antibodies which recognize and could hydrolyze QS-signals, as well as enzymes which cleave the QS-signals [6]. Moreover, entire organisms which exhibit QQ-capacity may be directly used as biocontrol agents [7]. The QQ investigations concern human, plant and animal health, as well as water engineering and anti-biofouling [6,8–11].

The N-acylhomoserine lactones (NAHLs) are QS-signals mainly produced by alpha-, beta-, and gamma-proteobacteria, including the pathogens *Agrobacterium tumefaciens*, *Burkholderia glumae*, *Pectobacterium atrosepticum*, *Pseudomonas aeruginosa*, *Pantoea stewartii* [3,4]. The QQ-enzymes degrading NAHL have been discovered in several species of Archaea, Eukarya and Bacteria [11]. These are lactonases which open the NAHL lactone ring, amidases which cleave NAHL molecules into homoserine lactone and fatty acids, and NAHL-modifying enzymes such as oxidases and reductases which alter the acyl chain [7]. In some QS-emitting pathogens such as *A*. *tumefaciens* and *P*. *aeruginosa*, QQ-lactonases and QQ-amidases are involved in the clearing and recycling of their own NAHL-signals [12–14]. Other bacteria, such as *Rhodococcus erythropolis*, *Bacillus thuringiensis* and *B*. *cereus*, do not produce NAHLs but are able to degrade them efficiently [15–17]. These QQ-organisms are proposed as biocontrol agents as well as reservoirs of quorum-quenching enzymes for biotechnology [8,9].

*R*. *erythropolis* is a unique actinobacterium in which three quorum-quenching activities using lactonase, amidase and reductase, have been discovered [18,19]. Consequently, several applied developments of the *R*. *erythropolis* quorum-quenching have been proposed in plant protection, anti-biofouling and water engineering [20–22]. In *R*. *erythropolis*, the lactonase QsdA has been identified as the only lactonase [19]. This enzyme belongs to the phosphotriesterase-like lactonases family and cleaves a broad spectrum of NAHLs [19]. To date, no genetic and biochemical information are available about the regulation of QQ-enzymes in *R*. *erythropolis*. To our knowledge, the only known transcriptional factor controlling QQ-enzyme expression is BlcR (AttJ) in *A*. *tumefaciens* [12].

In this work, genome engineering is proposed for improving quorum-quenching capabilities of *R*. *erythropolis*, and to access functional and structural characterization of transcriptional regulators controlling the QQ-pathway. Using directed evolution, we selected *R*. *erythropolis* derivatives in which QS-signal degradation capability were improved in comparison with the parental strain *R*. *erythropolis* R138. We then combined deep-sequencing, molecular and structural biology for identifying and characterizing the incriminated mutations. This study highlights that a single nucleotide variation in key-transcriptional factors is enough for improving functional properties of QS-signal degrading organisms and that directed evolution may be used to understand regulatory pathways of interest in bacteria which are recalcitrant to genetic manipulations.

## Materials and Methods

### Selection of the *R*. *erythropolis* variants with an enhanced QS-signal assimilation

The wild type strain *R*. *erythropolis* R138 [23] was cultivated at 30°C in a synthetic AB medium [24], which is supplemented with ammonium chloride (1 g/L) and mannitol (2 g/L) as nitrogen and carbon source (AB-man). *N*-octanoylhomoserine lactone (C8HSL) and 3-oxo-octanoylhomoserine lactone (OC8HSL) from Sigma-Aldrich (St-Louis, MO, USA) were used as alternative carbon sources at 1 mM in AB-C8HSL and AB-OC8HSL media, respectively. A single pre-culture of the wild type strain *R*. *erythropolis* R138 was used for starting the propagation of three independent lineages in AB-OC8HSL. Twice a week, a fresh AB-OC8HSL medium was subsequently inoculated up to 7 weeks. After 7 and 14 subcultures, a single clone was isolated from each of the three lineages. The strain *R*. *erythropolis* R138 and its evolved derivatives were stored at -80°C.

### C8HSL and OC8HSL assimilation assay

The parental strain *R*. *erythropolis* R138 and its evolved derivatives (M7.1, M7.2, M7.3, M14.1, M14.2 and M14.3) were cultivated at 30°C in AB-man medium for 24 h, then cells were washed in NaCl (0.8%) and suspended in AB-C8HSL and AB-OC8HSL media. The OD_600_ measurements were carried out every day. At the end of the bacterial growth, C8HSL and OC8HSL were extracted and quantified according to a procedure adapted from Cha *et al*. [25]. Briefly, bacterial cell cultures were centrifuged for 10 min at 15,000 *g*, and NAHLs were extracted from the supernatant by addition of one volume of ethyl acetate and by further air-drying the organic fraction. The extracted NAHLs were dissolved in 20 μL of ethyl acetate, of which 5 μL was spotted on TLC (Thin Layer Chromatography) silica plates (Macherey-Nagel, Düren, Germany). TLC plates were overlaid with the NAHL-biosensor strain *A*. *tumefaciens* NT1(pZLR4) in AB medium supplemented with agar (15 g/L) and X-gal (40 μg/mL). For quantification, calibration curves were obtained with pure C8HSL or OC8HSL.

### Variant search

Genome sequencing of the *R*. *erythropolis* mutants M7.1, M7.2 and M7.3 was performed at the IMAGIF sequencing platform (CNRS, Gif-sur-Yvette, France) using Illumina Genome AnalyserIIx (paired-end, 2×74 bp reads) as described by Kwasiborski *et al*. [26]. Sequence reads obtained for the OC8HSL consumer mutants were mapped on the annotated reference genome of *R*. *erythropolis* R138. Mappings were carried out using the CLC Genomics Workbench v7.5 (CLC bio, Aarhus, Denmark) with a read length (90%) and similarity (95%). Genomic variant detection was processed using CLC Genomics Workbench with a variant occurrence of 100%. Characteristics of the evolved derivatives are described in Table 1.

**Table 1:** Characteristics of bacterial derivatives M7.1, M7.2 and M7.3.

Mutant	Gene name	Mutation position	Nucleotide variation	Amino acid position	Amino acid variation
M7.1	*qsaR*	787	C > A	263	Glu > Stop
M7.2	*qsdR*	133	C > A	45	Gly > Cys
M7.3	*qsaR*	692	G > A	231	Ser > Phe

### Quantitative RT-PCR

Gene expression was quantified by RT-qPCR using biological triplicates. Sequences and characteristics of the primers are presented in Table 2. Reverse transcriptions were carried out using the protocol for high GC content bacteria from the Revert Aid Reverse Transcriptase (Fermentas, Whaltham, USA). A Light Cycler 480 (Roche Applied Science, Penzberg, Germany) and Light Cycler 480 SYBR Green I Master (Roche Applied Science) were used for quantitative PCR. The 15 μL final volume mix contained SYBR Green I Master (1x), forward and reverse primers (1 μM) and 0.01 μg of cDNA samples. After denaturation at 95°C for 10 min, the amplification and quantification program was repeated 45 times as follows: 95°C for 15 s, 60°C for 15 s, 72°C for 20 s, with a single fluorescence measurement, followed by the melting curve program (65°C-95°C with a heating rate of 0.1°C/s and a continuous fluorescence measurement) and a final cooling step at 45°C. The recombinase A (*recA*) gene was used as a reference gene in order to normalize gene expression.

**Table 2:** Sequences and characteristics of primers used in quantitative RT-PCR.

Gene identifiant	Gene name	Primer	Sequence (5'-3')	Position on chromosome^a^	Product size (bp)
CDS3910	*recA*	recA-F	ACGGATATCGGTGTTCTCCA	4160344	206
		recA-R	CACTCGAGTCAAGGTCGTCA	4160550	
CDS1197	*qsdR*	qsdR-F	AGCGTGATCGTCAGTTGG	1261433	269
		qsdR-R	AATCGCGACGAACTGCTC	1261702	
CDS1198	*qsdA*	qsdA-F	ACGAGCATGTCTTCGTTCTG	1262077	144
		qsdA-R	GGATCGACGATCGTGCTGAT	1262202	
CDS1199	*qsdC*	qsdC -F	AGGTTGCACTCGGATACTGG	1264216	199
		qsdC -R	GGCAGGGTGTTCGTAGAGAA	1264396	
CDS1200	*qsdD*	qsdD -F	AAGCGGAACTCACTGCTCAT	1265773	198
		qsdD -R	TGACTGCGATGAAGAACAGC	1265952	
CDS816	*qsaR*	qsaR-F	TTGTGACGAGCGAATTGAGA	889122	249
		qsaR-R	GAAGTGACAGTGGGGACGAT	889352	
CDS819	*qsaA*	qsaA -F	ACTTCCGCTCTCTCAACGAC	891654	203
		qsaA -R	TTTCGTCCGATGTGTACTGC	891838	
CDS820	*qsaB*	qsaB -F	GGCTACACGTTCGACTCGTT	889886	216
		qsaB -R	AACTGCACACGCAGAAGATG	890083	

^a^ Nucleotide position is given according to genome sequence of *R*. *erythropolis* R138 (NCBI ASKF00000000).

### Expression and purification of *QsdR*_*wt*_ *and* QsdR_G45C_

QsdR_wt_ and QsdR_G45C_ nucleotide sequences were chemically synthesized using codon optimization for expression in *E*. *coli* and inserted into pET29b expression plasmid using NdeI and SacI restriction enzymes (Genscript, Piscataway, NJ). *E*. *coli* BL21 competent cells transformed with pET29b-QsdR_wt_ were grown in 2TY media at 37°C (initial OD_600_ of 0.1) until an OD_600_ of 0.6 reached within 3 hours. Expression was induced for 4h by addition of 0.5 mM of isopropyl β-D-1-thiogalactopyranoside. The cells were pelleted by centrifugation at 8000 g for 20 min at 4°C and stored at -20°C before being resuspended in buffer A (50 mM Tris-HCl pH 8, 150 mM NaCl) and 20 mM imidazole and sonicated. After centrifugation at 25000 g for 45 minutes, the filtered supernatant was injected on a nickel affinity column (HiTrap 5 mL, GE Healthcare). After a washing step with buffer A and 35 mM imidazole, the protein is eluted with buffer A and 300 mM imidazole before its injection on a gel filtration Superdex 200 26/60 (GE Healthcare) using buffer A. The protein fractions are pooled, concentrated using a 5,000 MWCO Vivaspin (GE healthcare) and stored at -80°C.

*E*. *coli* C41 cells transformed with the plasmid pET29b-QsdR_G45C_ were grown at 37°C in LB media until an OD_600_ of 0.5. The pelleted cells were resuspended in fresh LB media supplemented with 4% (v/v) of ethanol and grown for 1 h at 20°C before inducing the expression with 0.5 mM isopropyl β-D-1-thiogalactopyranoside for 16 h. The cells were pelleted by centrifugation at 8000 g for 20 min at 4°C and stored at -20°C. The purification protocol was the same as for QsdR_wt_ in presence or in absence of Dithiothreitol (DTT).

### Crystallization and data collection

Crystallization conditions for QsdR_wt_ at 16 mg/mL were screened using Qiagen kits (Valencia, CA, USA) with a Cartesian nanodrop robot (Genomic solutions). Two conditions manually optimized in hanging drops composed of a 1:1 volume ratio of protein solution and crystallization solution (20% 2-Methyl-2,4-pentanediol (MPD) or 20% Isopropanol, 0.2 M CaCl2, 0.1 M Na Acetate pH 4.5) led to crystals. Crystals from MPD conditions were directly flash-frozen in liquid nitrogen while those from isopropanol condition were transferred into mother liquor supplemented with 25% PEG 400 before. X-ray diffraction datasets were collected at 100 K on Proxima 1 beamline (SOLEIL synchrotron, Saint-Aubin, France). The datasets used for sulphur phasing were collected at λ = 1.7712 Å wavelength (7 keV) with an oscillation range of 0.1° and 0.1 s of exposure per image. Five datasets were collected: 360° around φ with κ = 0 and ω = 0, 180° around ω at φ = 0° and κ = 15°, 180° around ω at φ = 180° and κ = 15°, 180° around ω at φ = 0° and κ = -15° and 180° around ω at φ = 180° and κ = -15°. Data were processed with XDS package [27] and all datasets were then merged using XSCALE [27].

### Structure determination and refinement

The crystal structure of QsdR_wt_ was determined at 2.4 Å resolution by SAD method from sulphurs contained in the protein. Solvent content analysis using CCP4 (Collaborative Computational Project, Number 4) indicated the presence of one monomer in the asymmetric unit (AU). The positions of 8 sulphur atoms were found using SHELX suite program [28]. Phases were calculated using PHASER [29] and density modification was performed by PARROT (CCP4 suite). An initial model covering 90% of the QsdR_wt_ sequence was automatically built using BUCCANEER [30]. This initial model was used as a search model for molecular replacement to solve the structure of the higher resolution dataset (1.9 Å resolution) collected from a different crystal form. An iterative process of manual building in COOT [31] combined with refinement using BUSTER-2.10 [32] with NCS restraints and TLS groups (two molecules in asymmetric unit) was performed. Refinement details of the highest resolution structure are shown in Table 3. Molecular graphics images were generated using PyMOL (http://www.pymol.org).

**Table 3:** Crystallographic data and refinement parameters.

PDB code	4ZA6	Not deposited
Crystallization conditions	**A**: 20% MPD, 0.2M CaCl2, 0.1M Na Acetate pH 4.5	**B**: 20% Isopropanol, 0.2M CaCl2, 0.1M Na Acetate pH 4.5.
**Data collection**		
Space group	I4_1_	P6_1_22
a/b/c (Å)	91.6/91.6/145.2	87.01/87.01/141.89
α/β/γ (°)	90/90/90	90/90/120
mol/UA	2	1
Resolution (Å)	50–1.97 (2.09–1.97)	50–2.40 (2.46–2.40)
Total reflections	284103 (45322)	1448518 (98482)
Unique reflections	42157 (6768)	23522 (1737)
Completeness (%)	99.9 (99.3)	99.9 (98.5)
I/σi	12.44 (1.86)	37.12 (3.30)
CC_1/2_	99.9 (85.7)	100 (94.8)
Rsym (%)	8.6 (90.2)	9.6 (143.7)
**Phasing**	MR from Sulphur-SAD model	Sulphur-SAD model
**Refinement**		
R factor/ R free (%)	20.7 / 22.8	
Rmsd bond (Å) / angle°	0.009 / 1.03	
Mean B factor (Å^2^)		
protein	38.3	
solvent	50.5	

Values in parenthesis are those for the last shell; MR means Molecular replacement. CC_1/2_ = percentage of correlation between intensities from random half‐dataset (P. A. Karplus, K. Diederichs, Science 2012, 336, 1030–1033).

### Circular dichroïsm experiments

Circular dichroïsm in the far-UV region was performed using a spectropolarimeter (Jasco J-810) equipped with a water-cooled Peltier unit (Jasco circular dichroïsm spectrometer model J810). QsdR was concentrated at 8 mg.ml^-1^ (wild type), 9 mg.ml^-1^ (QsdR_G45C_) or 11 mg.ml^-1^ (QsdR_G45C_ +DTT) in 50 mM Tris pH 8 and 150 mM NaCl Spectra were recorded in a cell width of 0.01-mm path length (121.QS, Hellma) from 185 to 260 nm at 20°C. Three consecutive scans from each sample were merged to produce an averaged spectrum; the spectra were corrected using buffer baselines measured under the same conditions. Data were recorded in mdeg and converted as delta epsilon (Δε, M^−1^.cm^−1^). Secondary structure estimates were derived from the normalized spectra using the CDSSTR, SELCON3, CONTIN of the DICHROWEB server, or K2D3 [33,34].

### Mass spectrometry protein identification

The presence of the protein in both *R*. *erythropolis* QsdR_wt_ and QsdR_G45C_ strains was checked by mass spectrometry. 50 ml of LB was inoculated with a colony of *R*. *erythropolis* wild type or *R*. *erythropolis* M7.2 mutant. Bacteria were grown at 28°C for 48 h. The volume of culture corresponding to 1 OD_600_ (1.250 and 1.430 μL is centrifuged then the pellet is resuspended in 20μL of protein loading dye) was loaded on a SDS-PAGE. Bands corresponding to the apparent molecular weight of the protein were excised and subjected to in-gel enzymatic digestion in the Progest robot (Genomic Solutions) using standard conditions. After overnight tryptic digestion, peptides were extracted with 60% acetonitrile and 0.1% (v/v) formic acid. Acetonitrile was removed under vacuum and peptides were resuspended in 0.1% (v/v) formic acid prior to LC-MS/MS mass spectrometry analyses.

LC-MS/MS analyses were performed with the Triple-TOF 4600 mass spectrometer (ABSciex) coupled to the nanoRSLC system (Thermo Scientific) equipped with a trap column (Acclaim PepMap100C18, 75 μmi.d.× 2 cm, 3 μm) and an analytical column (Acclaim PepMapRSLCC18, 75 μmi.d.× 25 cm, 2 μm, 100 Å). Peptides were eluted at a flow rate of 300 nl/min from the reverse phase C18 column using a 5–35% CH_3_CN gradient for 40 min. MS/MS spectra were acquired with a Data Dependent acquisition method by selecting the 20 most intense precursors for CID fragmentation. Raw data were analysed with PeakView software (ABSciex) and processed with MS Data Converter software for generating.mgf data files. Protein identification searches were performed using the MASCOT algorithm and nrNCBI database considering cysteine carbamidomethylation as complete modifications and oxidation (methionine and tryptophan) as variable modifications; peptide and fragment tolerance were respectively set at 10 ppm and 0.01 Da. Only ions with a score higher than the identity threshold at less than 1% of false positive discovery rate (<1% false discovery rate using the decoy option in Mascot) were considered.

### Isothermal titration microcalorimetry measurements

Isothermal titration microcalorimetry experiments were performed with an ITC200 isothermal titration calorimeter from MicroCal (GE Healthcare). The experiments were carried out at 20°C. Protein concentration in the microcalorimeter cell (0.2 ml) was 25 μM. 19 injections of 2 μl of putative effectors solution (OC8-HSL, C8-HSL, 4-hydroxybutanoic acid lactone and gamma-caprolactone) with a concentration of 250 μM were performed at intervals of 180 s while stirring at 1000 rpm.

## Results

### Directed evolution improved OC8HSL degradation capability of *R*. *erythropolis* R138

**Fig 1:**
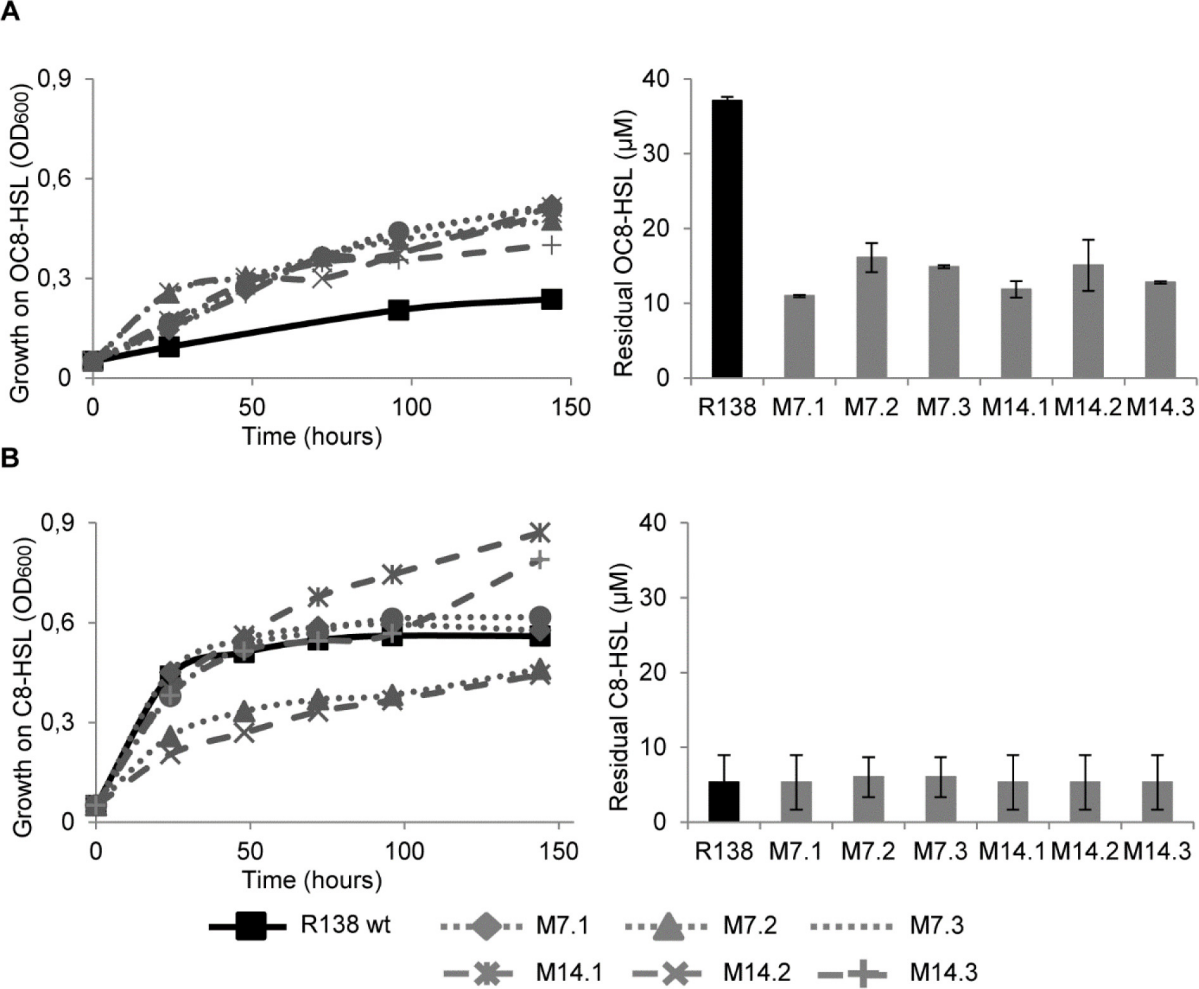
Assimilation and degradation of quorum-sensing signals. Growth of *R*. *erythropolis* R138 wt and its evolved mutants M7.1, M7.2, M7.3, M14.1, M14.2 and M14.3 in the presence of OC8HSL (A) and C8HSL (B) as a sole carbon source. Left panels show growth curves (OD_600_), right panels indicate concentration of residual quorum-sensing signals at the end of the growth (140 hours post-inoculation).

*R*. *erythropolis* R138 wild-type grows much better in a minimal medium supplemented with C8HSL than with OC8HSL (Fig 1), suggesting that the degradation and assimilation of QS-signal exhibiting a keto substitution at the carbon-3 in the acyl chain is limited.

Sub-cultures of the parental *R*. *erythropolis* R138 in three parallel lineages on the minimal medium supplemented with OC8HSL led to the isolation of six clones M7.1, M7.2, M7.3, M14.1, M14.2 and M14.3. These clones were named according to the sampling time (7^th^ and 14^th^ subcultures) and the lineage (1, 2 and 3). Growth of the parental *R*. *erythropolis* R138 and its derivatives was compared. After 144 h of incubation in the AB-OC8HSL medium, all evolved derivatives reached a higher culture density (OD_600_ = 0.4–0.5) compared with that (OD_600_ = 0.2) of the parental strain R138 (Fig 1A). This increased growth which is correlated with a decreased of the residual OC8HSL in the culture medium (Fig 1A) indicates that a better assimilation of OC8HSL occurs in all evolved derivatives. Residual NAHLs are those which are not altered by QQ-enzymes, irrespectively of their use as a nutrient.

In contrast, the evolved derivatives (except M7.2 and M14.2) and their parent *R*. *erythropolis* R138 grow similarly on C8HSL as a sole carbon source (Fig 1B). At the end of the growth assay (140 hours), the concentration of residual C8-HSL was similar in all culture media (Fig 1B).

### Genomic characterization of evolved derivatives M7.1, M7.2 and M7.3.

As no improvement of the OC8HSL-assimilation was observed in the clones collected after the 14^th^ subculture compared with clones of the 7^th^ (Fig 1), we focused on the earliest derivatives M7.1, M7.2 and M7.3. Their total DNA was extracted and sequenced by Illumina technology using libraries of 300 bp fragments of which both extremities were sequenced. The number of filtered reads reached 20 267 452, 18 155 476 and 22 655 016 for clones M7.1, M7.2 and M7.3, respectively. All of the reads were mapped on the genome sequence of the parental strain *R*. *erythropolis* R138 [26] with a mean coverage ranging from 193 to 240.

Using the CLC software and a selective filter at 100%, only three independent non-synonymous substitutions were identified on the circular chromosome (Table 1). In the M7.2 derivative, the mutation 7.2 is located in the gene CDS1197 which is adjacent to the *qsdA* gene (CDS1198) encoding the known NAHL cleaving lactonase QsdA [19]. We named the incriminated gene *qsdR* (quorum-sensing degradation regulation) which codes for a transcriptional regulator of the TetR/FabR family. In the M7.1 and M7.3 derivatives, the distinctive mutations belong to the same gene CDS816, encoding a transcriptional regulator of the RipR family. We call this gene *qsaR* (quorum-sensing assimilation regulation).

### qsd and qsa clusters were overexpressed in the evolved derivatives—In *R*. *erythropolis*

**Fig 2:**
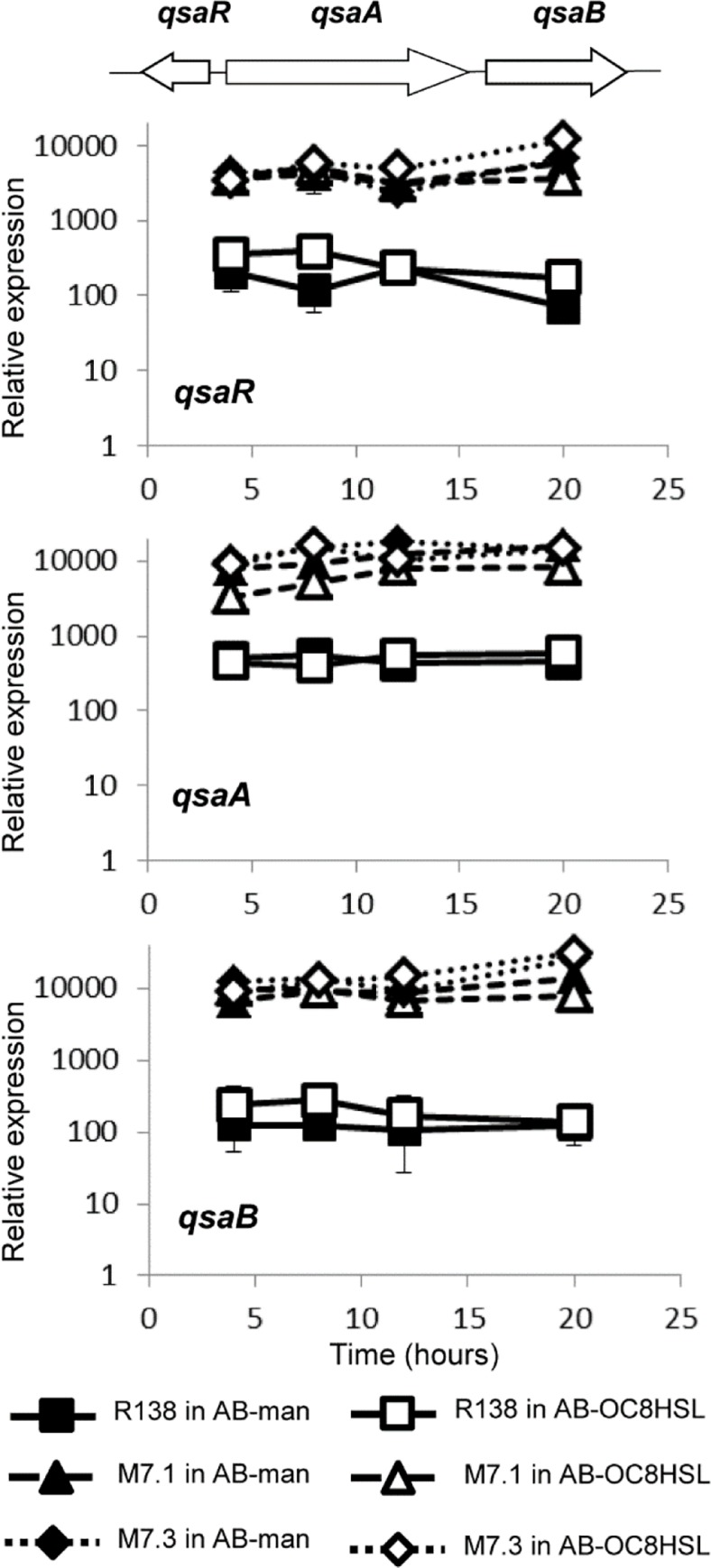
Expression of the *qsaRAB* genes. RT-qPCR monitoring of the *qsaRAB* expression in *R*. *erythropolis* R138 wt and its mutants M7.1 and M7.3 grown in the presence of mannitol (AB-man) and OC8HSL (AB-OC8HSL) as a sole carbon source. Expressions were normalized using the *recA* gene as a reference gene. Experiments were done in triplicate.

R138 wild-type, the gene *qsaR* is divergently transcribed from two adjacent genes, that we named *qsaA* and *qsaB*, coding for an amidohydrolase (CDS819) and a transporter (CDS820) of the Major Facilitator Superfamily (MFS), respectively. In the wild-type strain R138 and its derivatives M7.1 and M7.3, the expression of genes *qsaR*, *qsaA* and *qsaB* was monitored by RT-qPCR in the presence of mannitol or OC8HSL as a sole carbon source (Fig 2). All the genes exhibited a higher transcription level in the evolved backgrounds as compared to that observed in the wild-type strain whatever the culture medium.

**Fig 3:**
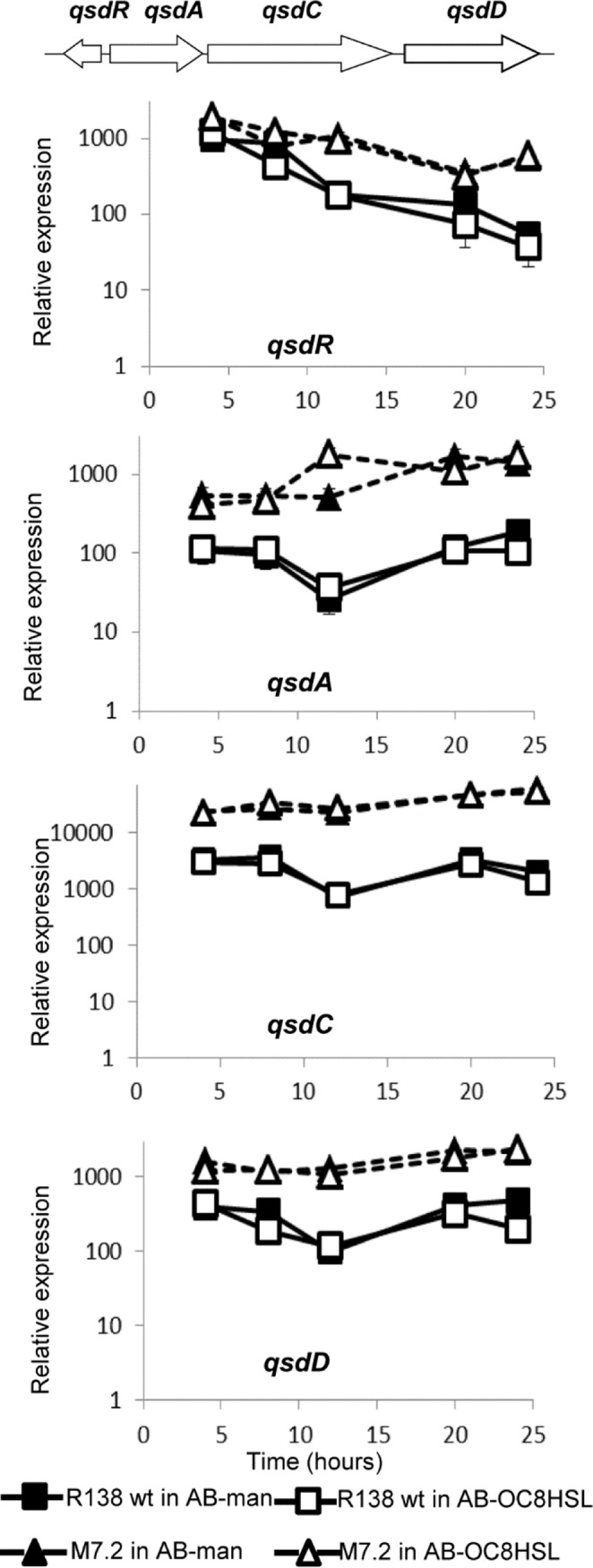
Expression of the *qsdRACD* genes. RT-qPCR monitoring of the *qsdRACD* expression in *R*. *erythropolis* R138 wt and its mutant M7.2 grown in the presence of mannitol (AB-man) and OC8HSL (AB-OC8HSL) as a sole carbon source. Expressions were normalized using the *recA* gene as a reference gene. Experiments were done in triplicated.

In *R*. *erythropolis* R138 wild-type, the gene *qsdR* is divergently transcribed from *qsdA* (the lactonase-coding gene) which is adjacent to two other genes coding for a long-chain fatty acid CoA ligase (CDS1199) and a MFS transporter (CDS1200). We called these two genes *qsdC* and *qsdD*, respectively. We did not use the name *qsdB* which was previously proposed for a QS-signal degrading amidohydrolase [35]. Expression of the *qsd* genes was compared between the wild type strain and the clone M7.2 in the presence of mannitol or OC8HSL as a sole carbon source. All the *qsd* genes were over-expressed in the clone M7.2 whatever the culture medium (Fig 3). In the wild-type and clone M7.2, the *qsdR* expression decreased by 90% in the course of the culture. As the lactonase QsdA is the best known QS-signal degrading enzyme in *R*. *erythropolis* [19,36], we thereafter studied its transcriptional regulator QsdR_wt_ and its variant QsdR_G45C_ (from M7.2).

### Structure and overall fold of QsdR

**Fig 4:**
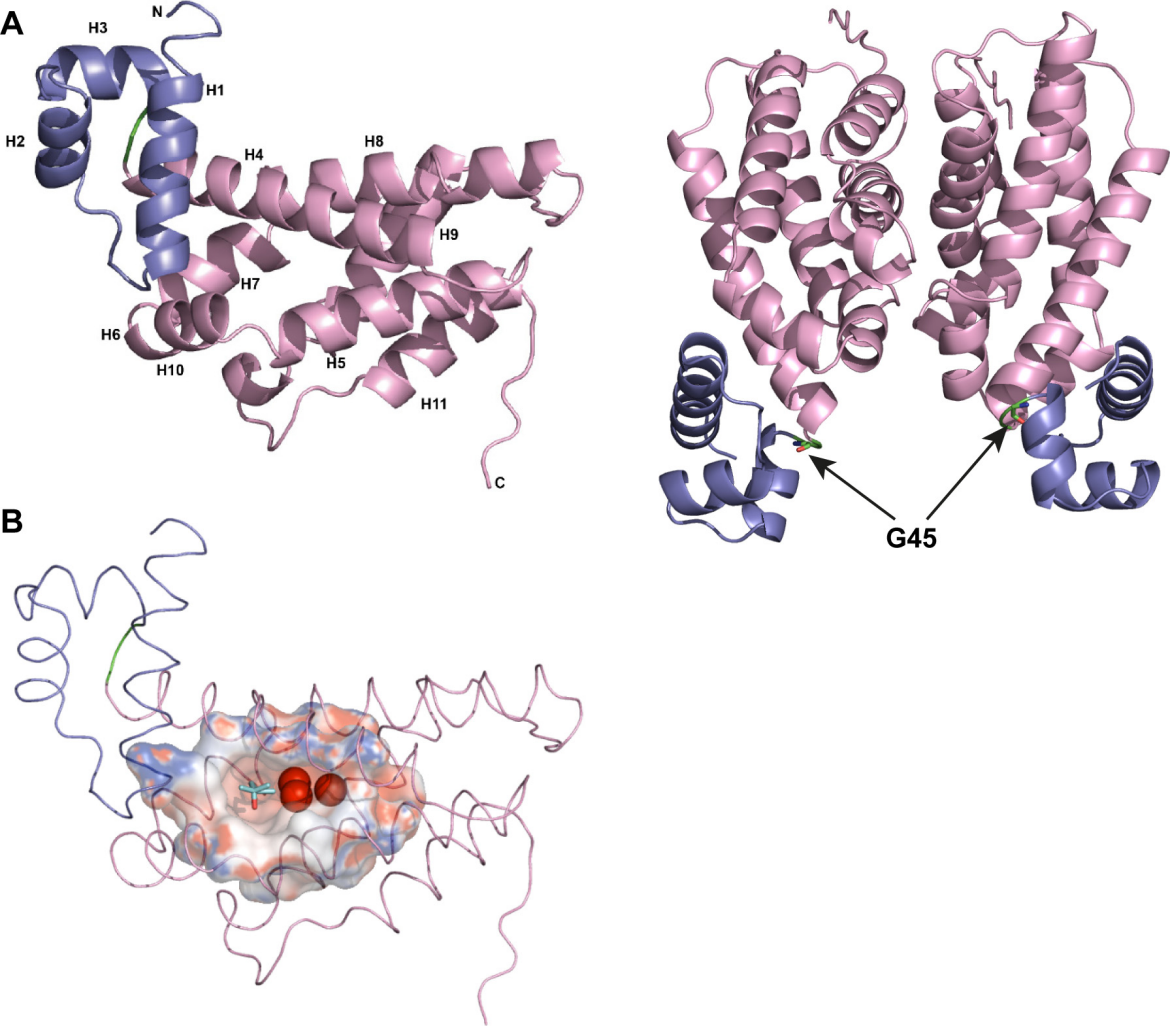
Structure of QsdR_wt_. (A) Ribbon representation of QsdR_wt_ fold. Its N-domain (residues 2–44) and C-domain (residues 47–186) are shown in blue and pink respectively. The linker between the two domains composed of Gly45 and Asn46 is in green. α helices (H) are numbered.(B) The putative binding pocket of QsdR_wt_. The protein is represented as trace and coloured blue for the N-domain (DNA binding domain) and pink for the C domain (effector binding domain). The cavity surface is shown in its electrostatic surface potential map. Red, blue and white colours correspond to negative, positive and neutral charged regions, respectively. The MPD molecule bound to QsdRwt and the bound water molecules are shown in cyan sticks and as red spheres respectively.

QsdR shares low sequence identity (around 20%) with regulatory proteins with known three dimensional structures. Thus the structure of QsdR at 2.4 Å resolution was solved using sulfur SAD method. One molecule was present in the asymmetric unit. A better resolution structure at 1.9 Å of QsdR from a different crystallization condition was determined by molecular replacement (using the sulfur-SAD structure as model) with two identical molecules in the asymmetric unit (root mean square deviation (rmsd) of 0.07 Å for 181 Cα atoms). Each molecule of QsdR_wt_ comprises 186 residues (Fig 4A) and is composed of 11 α helices, 3 of them corresponding to the DNA-binding domain, and the others to the regulatory domain. The DNA-binding domain (residues 2 to 41) forms the N terminus domain while the regulatory domain (residues 47 to 186) constitutes the C terminus. These two domains are connected by two residues, Glycine 45 and Asparagine 46. Each monomer in the asymmetric unit forms a dimer by the crystallographic symmetry (Fig 4B). The dimer interface covers 995 A^2^ per subunit involving 19 residues located in helices α7, α 9 and α10 and two loops, one between α7 and α8 and the other between α9 and α10. Therefore, the dimeric structure of QsdR_wt_ is the functional form in solution in line with results from gel filtration chromatography (molecular mass estimates at 44 kDa).

Although QsdR protein belongs to the Helix-Turn-Helix superfamily of regulatory proteins, a structural comparison of QsdR_wt_ with all PDB entries using SSM-EBI (http://www.ebi.ac.uk/mrd-srv/ssm) [37] shows a very low structural similarity with known structures. The lowest rmsd value of 2.81 Å was obtained with the *Thermus thermophilus* fatty acid degradation transcriptional repressor FadR, of which the structure was solved in presence of a bound dodecyl-CoA [38]. Therefore, we cannot infer any putative regulatory molecule for QsdR based on structural similarity. However, a bound MPD molecule from the precipitant solution well defined in electron density maps indicates the entrance of a protein cavity and forms three hydrogen bonds with the OH side chains of Tyr18 and Thr59 and the CO main chain of Leu55. The cavity contains 3 deeply buried water molecules (Fig 4B). While two water molecules directly interact with protein residues, the third one is bound to one of these water molecules. The cavity which is surrounded by α helices is formed by 21 residues: Tyr18 from α helix 1 (H1), Leu55, Thr59 and Tyr63 belonging to H4, Phe 82, Val85, Met86, Ser88, Val89 from H5 Ser92 from the loop between H5 and H6, Leu95 from H6, Phe106 and Ala110 from H7, Ile116, Glu117, Ser120 from H8 Val151, Cys154 and Asp155 from H9 Leu158 and Tyr159 from the loop between helices 9 and 10. This deep cavity interior is mainly polar while the entrance contains hydrophobic residues. The size of the putative regulator binding site excludes the accommodation of an effector bound to a CoA like the effector of FadR.

### Determination of NAHL/QsdR possible affinity

We used isothermal titration microcalorimetry to measure a possible affinity between QsdR_wt_ and several putative effectors: OC8HSL, C8HSL, gamma-butyrolactone and gamma-caprolactone. All these compounds exhibit a gamma-lactone ring and are substrates of QsdA [39]. No interaction was detected between QsdR_wt_ and any of these four molecules.

### Fold characteristics of the QsdR_G45C_ of the derivative M7.2

QsdR_G45C_ protein was purified using the same protocol as for QsdR_wt_. However, the size exclusion chromatography elution profile was different because the major fraction of the protein was eluted in the exclusion volume of the column indicating that QsdR_G45C_ was mostly aggregated. Nevertheless, a small remaining protein fraction was eluted (Data not shown). Despite several crystallization attempts, obtaining crystals of QsdR_G45C_ was unsuccessful. In contrast to QsdR_wt_, QsdR_G45C_ seems instable.

**Fig 5:**
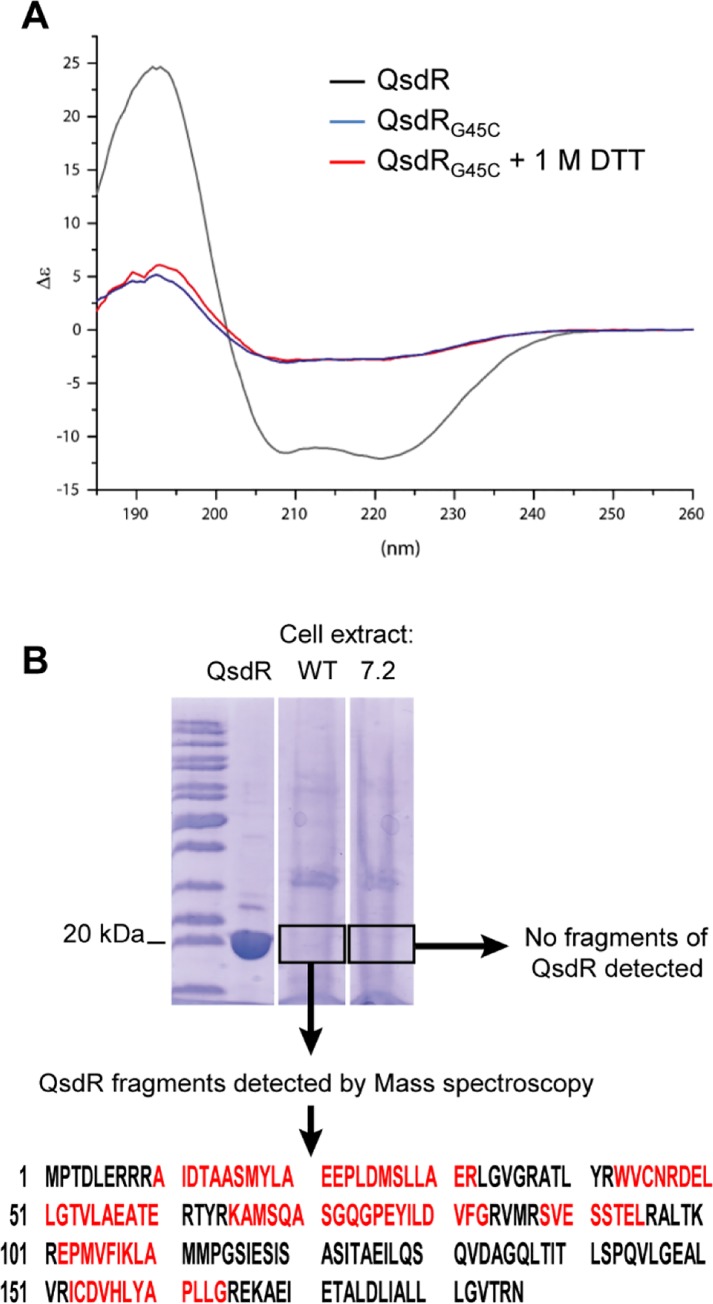
In vitro and in vivo stability of QsdR_G45C_. (A) CD analysis spectra of QsdR (black), QsdR_G45C_ (red) and QsdR_G45C_ in presence of DTT (blue). (B) Mass spectrometry analysis from a 12.5% SDS PAGE. Lane 1 control: purified QsdR protein; lane 2: whole protein content of wild type *R*. *erythropolis* R138; lane 3: whole protein content *R*. *erythropolis* mutant M7.2. Protein bands around the corresponding gel area of pure QsdR were digested by trypsin and identified by LC-TOF/TOF peptide mass fingerprinting searching for matching fragments (Matched peptides are shown in red).

In order to determine and compare the secondary structures of QsdR_wt_ and QsdR_G45C_, each protein was analyzed by circular dichroïsm. QsdR_wt_ displays a high proportion of α helices (~ 91%) in line with what is observed in the crystal structures. In contrast, QsdR_G45C_ presents only 25% of α-helix secondary structures (Fig 5A and Table 4). One more analysis was performed with QsdR_G45C_ purified in presence of 1 mM DTT to prevent the formation of a disulphide bond between the unique cysteine of the protein and that additional introduced by the M7.2 mutation. The drastic loss of α-helix secondary structures was not recovered in presence of DTT proving that the 7.2 mutation is responsible for an improper folding of QsdR_G45C_.

**Table 4:** Secondary structure estimations from CD experiments.

	QsdR_wt_	QsdR_G45C_	QsdR_G45C_+DTT
**α-helices**	91%	29%	26%
**β-sheets**	2%	21%	22%
**Random coil**	6%	50%	52%

### Comparative accumulation of QsdR in *R*. *erythropolis* R138 wt and its derivative M7.2

The presence of QsdR_wt_ and QsdR_G45C_ in *R*. *erythropolis* R138 and its derivative M7.2 respectively was checked by mass spectrometry analysis from the whole cells proteins content. The same amount of bacterial cells for wild-type and clone M7.2 was loaded on a SDS-PAGE. Proteins in the gel area corresponding to the molecular weight of QsdR were analyzed by LC-MS/MS after trypsine digestion and fragments corresponding to QsdR_wt_ and QsdR_G45C_ were searched. As shown in Fig 5B, fragments of QsdR_wt_ were detected in wild type strain whereas none was detected in the M7.2 derivative. This suggests that the entire QsdR_G45C_ transcriptional factor is absent in the M7.2 bacterial cells.

## Discussion

In this work, we used genome engineering as a tool to improve the QS-signal degradation capacity of the bacterium *R*. *erythropolis*, which is a biocontrol and anti-biofouling agent [20–22,40]. The selective process was successful as it generated three evolved derivatives (M7.1, M7.2 and M7.3) exhibiting a higher assimilation of 3-oxo-subsituted QS-signals compared with their ancestor. This acquired function is of primary interest as the 3-oxo-subsituted QS-signals are produced by several pathogenic and biofilm-forming bacteria [3,4]. In previous works, directed mutagenesis and directed evolution were used for increasing NAHL production by NAHL-synthase LuxI [41], for modifying selectivity of the NAHL-sensor LuxR [42] as well as that of the NAHL-degrading amidase PvdQ [43]. Our work demonstrates that NAHL-degradation metabolic network may be enhanced in an entire organism by selecting mutations in key-regulators, even if they were previously uncharacterized. In addition, our attempts to construct mutants by reverse genetics in *R*. *erythropolis* R138, were unsuccessful, hence the approach based on natural selection was a helpful alternative way for identifying and studying the key-regulatory transcription factors involved in NAHL-degradation.

All the identified mutations are single nucleotide polymorphisms (SNPs) in genes coding for two transcriptional factors, revealing them as master-regulators of QS-signal degradation in *R*. *erythropolis*. In the derivatives M7.1 and M7.3, two different SNPs were located in the same gene (*qsaR*) coding for a transcriptional regulator of the RipR family and controlling the expression of putative amidase and MSF-transporter. The functions of these genes remain to be characterized in further work. In the derivative M7.2, the *qsdR* gene codes for a transcriptional regulator of the TetR/FabR family, which is adjacent to the *qsdA* gene encoding a QS-signal cleaving lactonase [19,44] as well as *qsdC* and *qsdD* genes coding for a putative long-chain fatty acid CoA ligase and a MSF-transporter, respectively. According to genome data of *R*. *erythropolis* PR4, QsdR has previously been proposed to regulate the expression of QsdA [44] which is able to cleave OC8HSL [39]. However, transcriptomics [45] and RT-qPCR (this work) showed that the expression of *qsdA* gene is not enhanced in the presence of OC8HSL in the culture medium. But, in the OC8HSL-assimilating mutant M7.2 the *qsdA* expression is higher than in the wild type ancestor. All together, these observations suggest that the level of expression rather than the catalytic properties of the lactonase QsdA seems a limiting factor for OC8HSL assimilation in *R*. *erythropolis*. Genome engineering allowed to overcome this limitation by selecting a bacterial derivative containing a single point mutation, G45C, in QsdR, exhibiting a QsdR-independent expression of QsdA. Transcriptional regulators appeared as recurrent targets for improving metabolic properties of microbes, including resistance to toxic compounds such as alcohols [46], production of metabolites such as fatty acids [47], and assimilation of metabolites [48].

QsdR protein possesses a DNA-binding domain at the N-terminus and a regulatory domain at the C-terminus. The DNA-binding domain permits the protein to bind DNA inducing genes activation or repression under its control. The two domains are linked by two residues including glycine 45 which was the mutated residue. Glycine is known to be highly flexible and its presence in this short linker can help the mobility between the two domains. Replacing this glycine by a cysteine (G45C) would have made this linker rigid. A model of this mutation shows no clash or steric hindrance preventing the stability or correct folding of such mutant protein. Unexpectedly, we show here that this single mutation G45C is responsible for QsdR misfolding and the lack of the mutant protein in the total protein pool of the bacteria although its corresponding gene is transcribed. These findings suggest that QsdR is rapidly degraded due to stability and fold problems, resulting in the constitutive expression of QsdA protein.

The regulatory domain of QsdR presents a half hydrophobic/half polar cavity suggesting that QsdR can bind molecules having both hydrophobic and polar groups such as NAHL. However, our results from the isothermal titration microcalorimetry did not reveal any affinity with the tested lactone ring-based molecules such as C8HSL or OC8HSL. Despite several co-crystallization attempts with NAHL and soaking crystals of QsdR_wt_, obtaining crystals of QsdR_wt_ in complex with NAHL was unsuccessful. Moreover, the cavity structure of the QsdR_wt_ regulatory domain has no similarity with that of the known structures of the TetR/FabR family such as FadR, a fatty acid degradation transcriptional repressor in *Thermus thermophilus* [38]. Therefore, we expect a difference in nature and size of the effector between these two transcriptional repressors. FadR is regulated with a CoA-link molecule whereas QsdR should be activated by a molecule without any CoA extension.

In conclusion, this work reveals that a single modification of only one amino-acid in a transcriptional factor leads to the creation of a new targeted genetic circuit in *R*. *erythropolis*. Hence, genome engineering based on natural selection appeared a powerful approach for identifying master-regulators in QS-signal degradation pathway, as well as for improving this pathway in QS-signal degrading organisms.

## Abbreviations

QQ: Quorum quenching
QS: Quorum sensing
NAHL: N-acyl-homoserine lactone
SAD: Single wavelength anomalous dispersion
